# Supplementary material for: Licorice Extract Isoliquiritigenin Protects Endothelial Function in Type 2 Diabetic Mice
Source: Nutrients. 2024 Sep 19;16(18):3160. doi: 10.3390/nu16183160 (PMC11435099; doi:10.3390/nu16183160)
Supplement: Supplementary file 1 [file nutrients-16-03160-s001.zip › nutrients-3189712-supplementary.pdf]

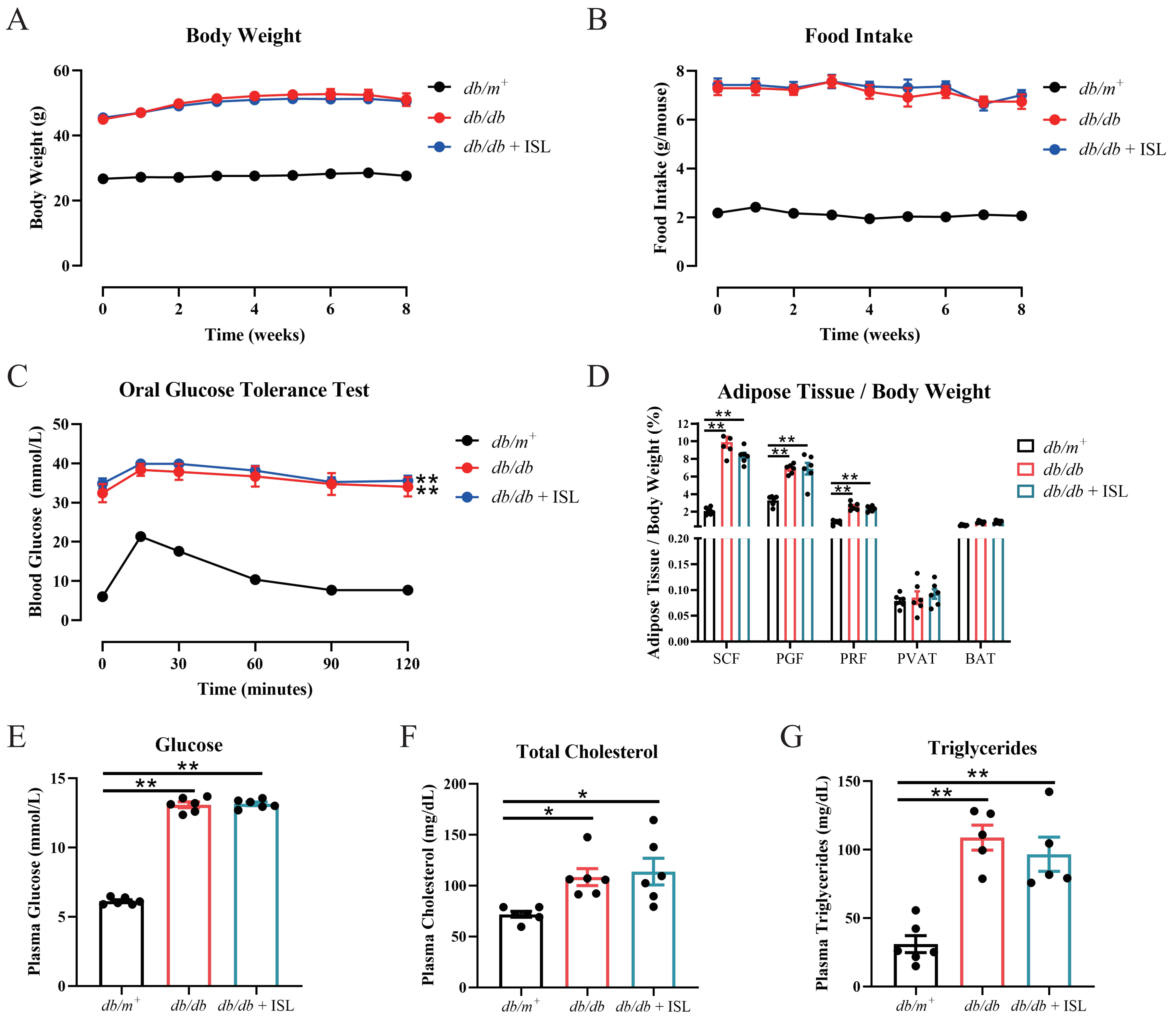

A
Body Weight
Body Weight (g)
Time (weeks)
db/m+
db/db
db/db + ISL
B
Food Intake
Food Intake (g/mouse)
Time (weeks)
C
Oral Glucose Tolerance Test
Blood Glucose (mmol/L)
Time (minutes)
D
Adipose Tissue / Body Weight
Adipose Tissue / Body Weight (%)
SCF
PGF
PRF
PVAT
BAT
E
Glucose
Plasma Glucose (mmol/L)
F
Total Cholesterol
Plasma Cholesterol (mg/dL)
G
Triglycerides
Plasma Triglycerides (mg/dL)

Supplement Figure Legend

Supplement Figure S1. Effects of oral ISL treatment on the basic parameters of *db/m*$^+$ and *db/db* mice. (**A**) Body weight and (**B**) food intake of *db/m*$^+$ and *db/db* mice treated and/or non-treated with ISL (20 mg/kg/day) via oral gavage for 8 weeks. (**C**) The Oral glucose tolerance test was performed at the end of ISL treatment. (**D**) Adipose tissue weight of *db/m*$^+$ and *db/db* mice treated and/or non-treated with ISL (20 mg/kg/day). (**E**) Plasma glucose levels, (**F**) plasma total cholesterol levels and (**G**) plasma triglycerides levels of *db/m*$^+$ and *db/db* mice treated and/or non-treated with ISL (20 mg/kg/day). Data represent means ± SEM of 6 mice. *p* values were determined using Two-way ANOVA for (**C**) and One-way ANOVA for (**D**, **E**, **F** and **G**), $^{**}p < 0.01$ *db/db*+ISL or *db/db* vs *db/m*$^+$ (**C**). $^{*}p < 0.05$, $^{**}p < 0.01$ (**D**, **E**, **F** and **G**). BAT, brown adipose tissue; PGF, perigenital fat; Phe, phenylephrine; PRF, perirenal fat; PVAT, perivascular adipose tissue; SCF, subcutaneous fat.

Primer Sequence used in qPCR analysis

Primers were purchased from Tech Dragon Limited. Primer sequences were shown below:

**Target**		**Primer Sequence**
β-actin	Forward Primer	AAGGCCAACCGTGAAAAGAT
	Reverse Primer	CCTGTGGTACGACCAGAGGCATACA
HO-1	Forward Primer	GATAGAGCGCAACAAGCAGAA
	Reverse Primer	CAGTGAGGCCCATACCAGAAG

ICAM-1	Forward Primer	GTGATGCTCAGGTATCCATCCA
	Reverse Primer	CACAGTTCTCAAAGCACAGCG
IL-6	Forward Primer	TGGGGCTCTTCAAAAGCTCC
	Reverse Primer	AGGAACTATCACCGGATCTTCAA
IL-10	Forward Primer	ATAACTGCACCCACTTCCCA
	Reverse Primer	GGGCATCACTTCTACCAGGT
MCP-1	Forward Primer	AAAACACGGGACGAGAAACCC
	Reverse Primer	ACGGGAACCTTTATTAACCCCT
NOX2	Forward Primer	TGGCTCCACTGGGAATTGC
	Reverse Primer	CAAACCCGGCATCATGGGA
Nrf2	Forward Primer	TAGATGACCATGAGTCGCTTGC
	Reverse Primer	GCCAAACTTGCTCCATGTCC
SOD1	Forward Primer	AACCAGTTGTGTTGTCAGGAC
	Reverse Primer	CCACCATGTTTCTTAGAGTGAGG
TNF-α	Forward Primer	TAGCCCACGTCGTAGCAAAC
	Reverse Primer	GCAGCCTTGTCCCTTGAAGA
VCAM-1	Forward Primer	AGTTGGGGATTCGGTTGTTCT
	Reverse Primer	CCCCTCATTCCTTACCACCC
